# Morphology, Chemistry and Function of the Postpharyngeal Gland in the South American Digger Wasps *Trachypus boharti* and *Trachypus elongatus*

**DOI:** 10.1371/journal.pone.0082780

**Published:** 2013-12-06

**Authors:** Gudrun Herzner, Martin Kaltenpoth, Theodor Poettinger, Katharina Weiss, Dirk Koedam, Johannes Kroiss, Erhard Strohm

**Affiliations:** 1 Evolutionary Ecology Group, Institute for Zoology, University of Regensburg, Regensburg, Germany; 2 Research Group Insect Symbiosis, Max Planck Institute for Chemical Ecology, Jena, Germany; 3 Department of Animal Sciences, Federal Rural University of the Semi-Arid Region, Mossoro, Rio Grande do Norte, Brazil; CNRS, France

## Abstract

Microbes pose severe threats to animals as competitors or pathogens and strongly affect the evolution of life history traits like parental care. Females of the European beewolf *Philanthus triangulum*, a solitary digger wasp, provision their offspring with paralyzed honeybees and embalm them with the secretion from large postpharyngeal glands (PPG) that contain mainly unsaturated hydrocarbons. This coating changes the physico-chemical properties of the prey surface, causes a reduction of water condensation and retards growth of mold fungi. Here we examined the closely related South American genus *Trachypus*, which shows a life-history similar to *Philanthus*. We investigated whether *Trachypus* spp. also possess PPGs and embalm larval provisions. Using histological methods and 3D reconstructions we show that *Trachypus boharti* and *T. elongatus* possess PPGs that are similar to *P. triangulum* but somewhat smaller. The ultrastructure of the gland epithelium suggests that the gland content is at least partly sequestered from the hemolymph. Chemical analyses using gas chromatography / mass spectrometry revealed that both the cuticle and PPGs of *Trachypus* contain mainly unsaturated long-chain hydrocarbons. The gland of *T. boharti* additionally contains long-chain ketones. The hydrocarbons from the PPG of *T. elongatus* occurred on prey bees excavated from nests in the field but not on conspecific control bees. While the embalming only slightly elevated the amount of hydrocarbons on prey bees, the proportion of unsaturated hydrocarbons, which is crucial for the antifungal effect, was significantly increased. The *Trachypus* species under study possess PPGs that are very similar to the PPG of *P. triangulum* with regard to morphology, ultrastructure and chemistry. Moreover, we provide clear evidence that *T. elongatus* females embalm their prey, presumably as a means of prey preservation. The observed differences among *Trachypus* and *Philanthus* in gland size and prey embalming may have evolved in response to divergent ecological conditions.

## Introduction

Pathogenic and competing microbes prevail everywhere that animals live. This permanent threat has given rise to a remarkable diversity and complexity of animal adaptations to protect themselves, their offspring as well as their food against microbial attack [1-8].

 A solitary digger wasp, the European beewolf *Philanthus triangulum* Fabricius (Hymenoptera, Crabronidae), for example shows several intriguing adaptations to such kinds of threats. Female beewolves provision brood cells with paralyzed honeybees (*Apis mellifera*, Hymenoptera, Apidae) as food for their offspring [9]. In the subterranean nests the prey bees and the developing brood are exposed to a multitude of biotic and abiotic threats. To protect the provisions and their brood against infestation by microbes, *P. triangulum* females embalm their prey with large amounts of hydrocarbons (HCs) [7,10-13]. This prey embalming considerably increases the total amount of HCs on the prey bee surface as well as the proportion of unsaturated and shorter-chain HCs [10-12]. Such a change in the chemical profile may reduce the melting point of the total hydrocarbon mixture on the prey bee surface [14,15], in that it becomes more or less liquid and forms an oily, hydrophobic layer [10]. As a result, water condensation on the bees is reduced so that the growth conditions for fungi are impaired and fungus growth is significantly retarded [7,10,13]. A second benefit of the thick layer of HCs caused by the embalming is a reduction of water loss from the prey bees that helps the beewolf larva to resist desiccation under hot and dry conditions [12].

The HCs used for embalming are secreted from a large head gland, the postpharyngeal gland (PPG) [16-18]. This type of gland has long been known only from ants [19-21], until it has recently been described in *P. triangulum* [16,22] as well as in the emerald cockroach wasp *Ampulex compressa* (Hymenoptera, Ampulicidae) [23]. In all these taxa, the PPG contains mostly HCs (but see 22,24) that show a high congruence with the respective epicuticular hydrocarbon profile [17,18,20,23,25]. In ants, several functions have been suggested for the PPG (for a review see 19), with the crucial role in nestmate recognition being the most prominent [26-28]. The PPG in *A. compressa* was proposed to function as a HC storing organ [23].

The simple yet ingenious physical mechanism of prey embalming to fight fungi and desiccation has so far only been described for *P. triangulum*, but preliminary analyses of other *Philanthus* species show that they also possess PPGs and embalm their prey (Herzner, Kaltenpoth and Strohm, unpublished). The embalming of prey with a PPG secretion thus seems to be common in the genus *Philanthus*, which comprises about 140 species with a distribution in Europe, Africa, North America, and Asia [29,30]. 

**Figure 1:**
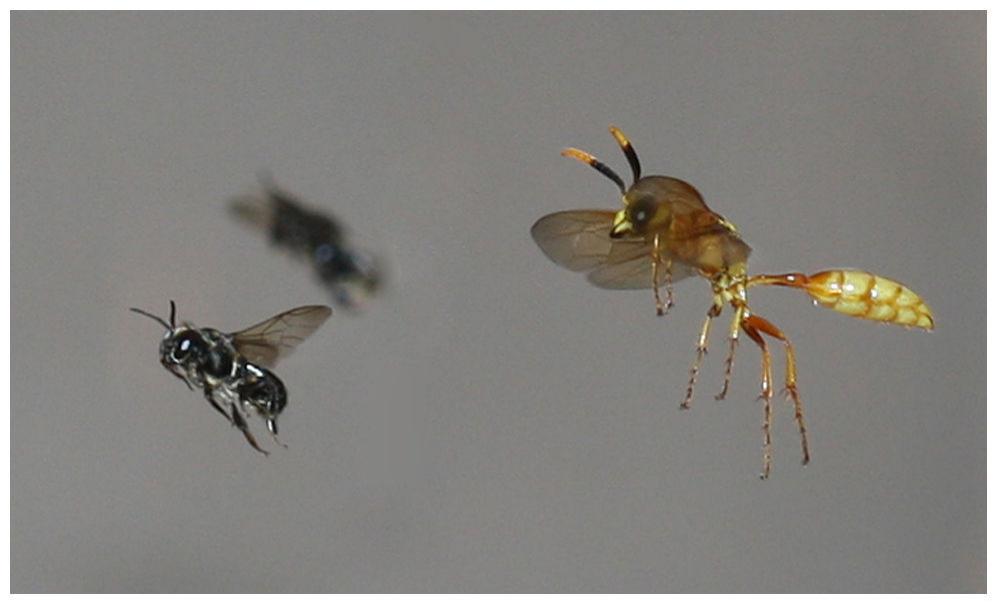
Female *T. boharti* hunting males of the stingless bee *Scaptotrigona postica* in flight.

Published morphological phylogenies [31] as well as a molecular phylogeny of the subfamily Philanthinae that is under construction (Kaltenpoth, Strohm et al., unpublished) suggest that the most closely-related taxon to *Philanthus* is *Trachypus*, a genus that comprises about 30 species in Central and South America [29,30]. The life-history of *Trachypus* is similar to that of *Philanthus*, with females constructing nests in the soil and hunting hymenopteran prey (Figure 1) as provisions for their progeny [30,32-34]. Thus, it is likely that *Trachypus*, as the ‘neotropical ecological equivalent to *Philanthus*’ [35], faces similar threats with regard to pathogenic and competing microorganisms and desiccation. 

We already know that *Philanthus* and *Trachypus* share another antimicrobial defense mechanism. European beewolves have evolved a protective symbiosis with *Streptomyces* bacteria (‘*Candidatus* Streptomyces philanthi’) that are cultivated in the females' antennae, secreted into the brood cells, taken up by the larvae and integrated into the cocoon [6,36,37] where they produce a mixture of antibiotics that fight a multitude of microorganisms and secure the survival of the developing beewolf [6,37,38]. Closely related bacteria have been found in the antennae of 28 other species of the genus *Philanthus* [36]. Recently, it has been shown that *Trachypus* as well as another related genus, *Philanthinus*, also engage in protective symbiosis with *Streptomyces* bacteria [39,40].

The present study aims to elucidate whether females of *Trachypus* spp. also possess PPGs and embalm their prey. We investigated two species, *Trachypus boharti* (Rubio) and *Trachypus elongatus* (Fabricius), for the presence, morphology, and ultrastructure of the PPG by light microscopy, transmission electron microscopy and 3D-reconstruction of cephalic structures based on semi-thin sections. The contents of the PPGs of the two *Trachypus* species were analyzed by gas chromatography – mass spectrometry to assess whether they are dominated by HCs. In addition, the chemical profiles of the cuticles were analyzed and checked for chemical congruence with the PPG content. To assess whether the prey in the brood cells is embalmed, provisioned prey bees were excavated from *T. elongatus* nests in the field, and their chemistry was compared with both conspecific control bees caught in the field and the chemical profile of the PPG of *T. elongatus* females. The results provide new insights into the evolutionary history of the PPG and the distribution and variation of embalming behavior within the crabronid wasps.

## Results

### Morphology and ultrastructure of *Trachypus* PPGs

**Figure 2:**
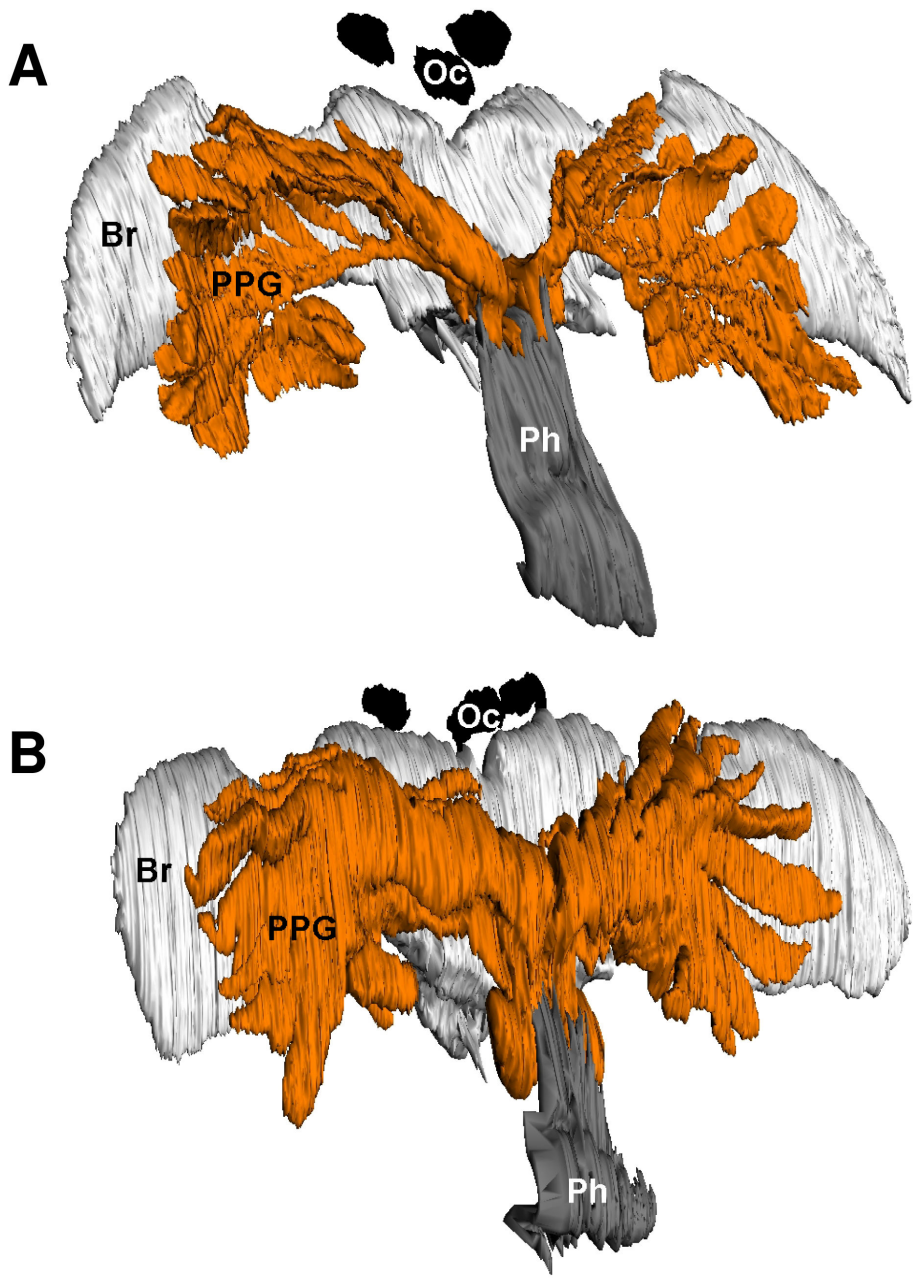
3D-reconstructions of the postpharyngeal glands of females. (**A**) *T. boharti*, and (**B**) *T. elongatus*. Abbreviations: Br, brain; Oc, ocellus; Ph, pharynx; PPG, postpharyngeal gland. Head capsule widths of the depicted individuals were 3.52mm for *T. boharti* and 3.64mm for *T. elongatus*.

The histological investigations revealed that females of both *T. boharti* and *T. elongatus* possess large gland reservoirs that originate from the pharynx near the posterior part of the hypopharyngeal plate and can thus be called PPGs. The 3D-reconstructions illustrate that the PPG is located in the forehead in the upper two thirds of the head capsule and extends laterally to the anterior margins of the eyes (Figure 2). The PPGs of both species originate from the pharynx and comprise various tubular extensions. In *T. elongatus*, the gland has an overall ‘glove-like’ structure (Figure 2B) with 10-12 ‘fingers’ originating from a common root. The PPG of *T. boharti*, in contrast, has a more ‘comb-like’ overall structure (Figure 2A) with 10-12 ‘teeth’ originating from a common branch. In *T. boharti*, the 'teeth' of the reconstructed individual appear somehow collapsed. The PPG of *T. elongatus* has an additional sac-like extension ventrally to the pharynx. The calculated gland volumes were 0.27 µl for *T. elongatus* (3.6 mm head capsule width) and 0.1 µl for *T. boharti* (3.5 mm head capsule width).

The histological investigations of the semithin sections revealed that in *T. boharti* the left and right ‘comb’ of the gland each have their own opening into the pharynx laterally near the suspensorium of the hypopharyngeal plate. In *T. elongatus*, in contrast, the roots of the two ‘gloves’ cohere and have one joint dorsal connection to the pharynx. In both species air sacs are located between the frontal cuticle and the PPG as well as between the PPG and the brain. 

**Figure 3:**
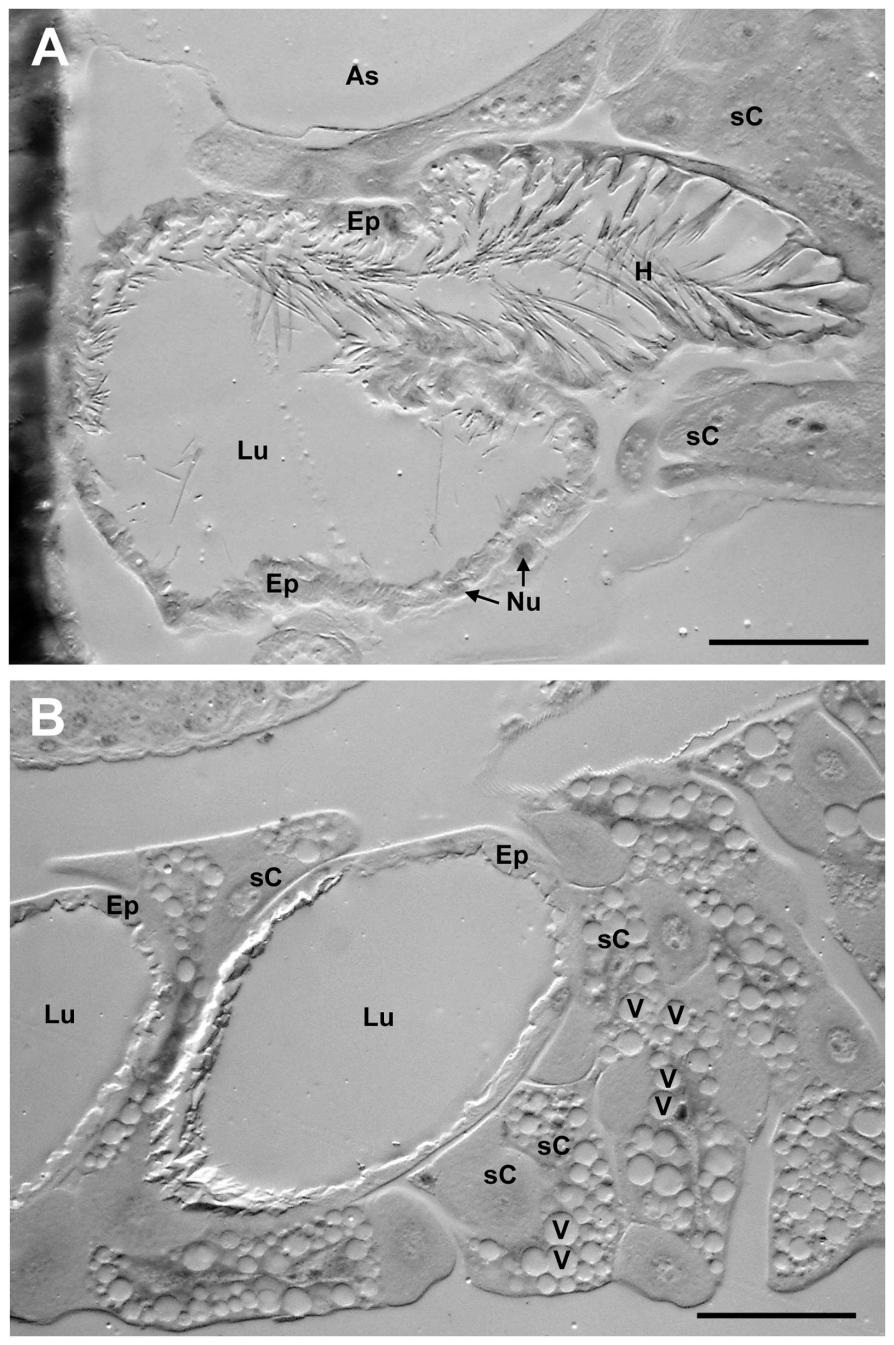
Semi-thin sections of the postpharyngeal glands of females. (**A**) *T. boharti*, and (**B**) *T. elongatus*. All the features shown for one species could also be observed in the other, but may not appear in the respective picture. Abbreviations: As, air sac; Ep, epithelium of the PPG; H, hair; Lu, lumen of the PPG; N, nucleus; sC, surrounding cell; V, vesicle (scale bars = 50µm).

In both species the wall of the PPG is formed by a monolayered epithelium (Figure 3). The basal (outer) side of the epithelium is characterized by multiple constrictions and foldings that apparently enlarge the outer surface. The apical (inner) side of the epithelium bears numerous long hairs that extend into the lumen. These hairs become less abundant towards the outer parts of the gland. There are no class-III gland cells [41,42] with duct cells and secretory cells associated with the PPG. In some regions the epithelium borders a tissue of relatively large cells, some of which contain numerous large vesicles (Figure 3). While there are muscles attached to the cuticular extensions of the hypopharyngeal plate near the opening of the PPG in both species, no muscles are directly associated with the epithelium of the gland. In *T. elongatus* there is a circular muscle around the opening of the lower sac-like part of the PPG.

**Figure 4:**
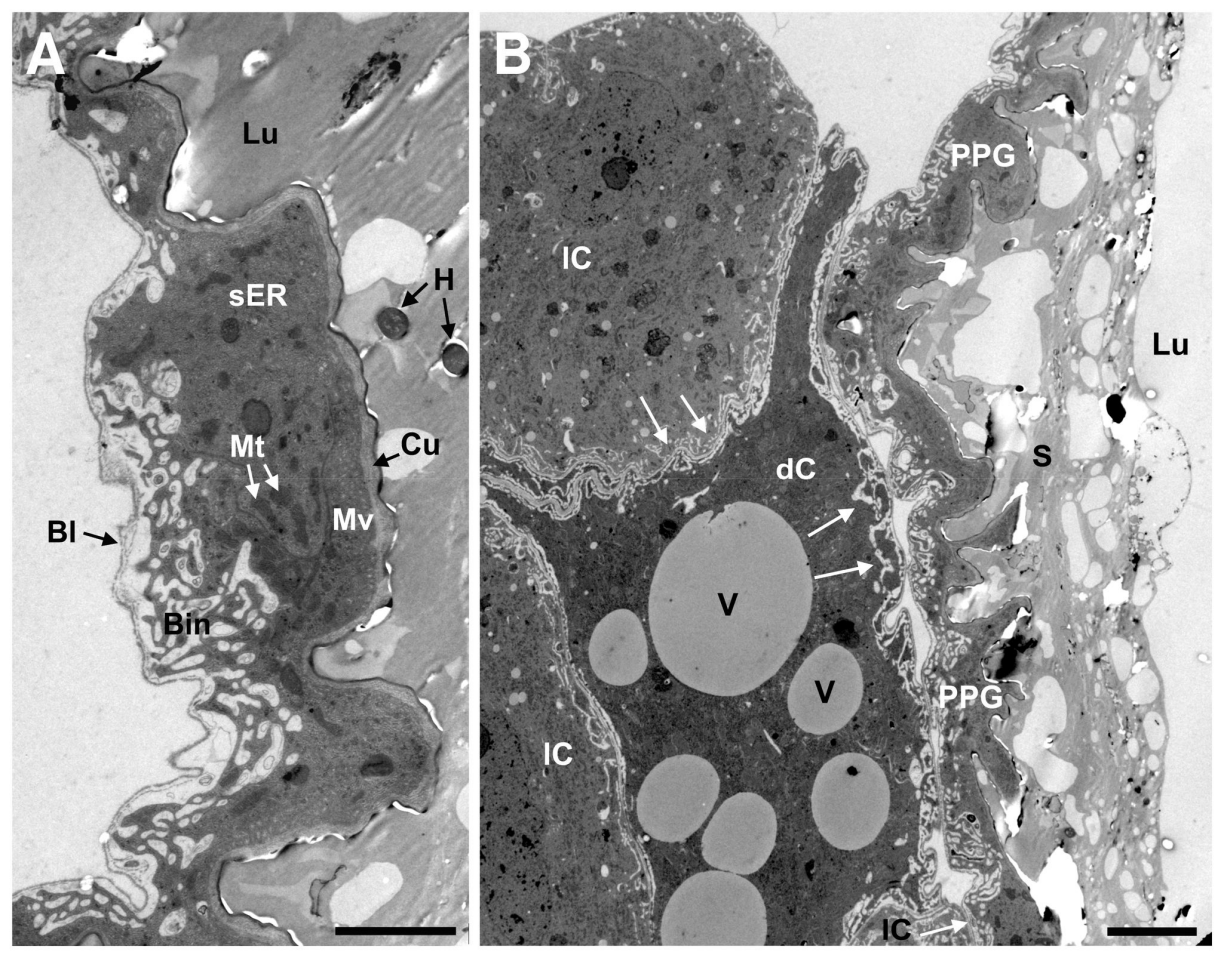
Transmission electron micrographs of the PPG epithelium of a female *T. boharti*. (**A**) Epithelium of the PPG (scale bar = 2.5µm); (**B**) PPG and the surrounding cells (scale bar = 5µm). The surrounding cells are in direct contact with the PPG and with each other (white arrows). Abbreviations: Bl, basal lamina; Bin, basal invagination; Cu, cuticle; dC, dark cell; H, hair (cross-cut); lC, light cell; Lu, lumen; Mt, mitochondrium; Mv, microvilli; PPG, postpharyngeal gland; S, secretion; sER, smooth endoplasmatic reticulum; V, vesicle.

The transmission electron microscopic investigations of the ultrathin sections of *T. boharti* PPGs confirmed that the wall of the gland reservoir is formed by a monolayered epithelium (Figure 4A). The thickness of this epithelium is rather variable. The apical side of the epithelium is characterized by a well-developed microvillar fringe and is supported by an intima (cuticle) that resembles the intima of the pharynx. The basal side of the epithelium is bordered by a basal lamina that shows numerous conspicuous invaginations. The epithelial cells contain large nuclei, mitochondria, smooth and some rough endoplasmatic reticulum, and septate desmosomes. There are no extracellular cavities, pore canals, or ducts that are characteristic of class-III gland cells [41].

The cells bordering the PPG in *T. boharti* seem to comprise two different types (Figure 4B). One type appears lighter and contains mainly smooth endoplasmatic reticulum in high densities as well as mitochondria. The darker appearing cell type contains mainly rough endoplasmatic reticulum, mitochondria and various large electron translucent vesicles (Figure 4B). Both cell types are found in close proximity with each other and the epithelium of the PPG. The pronounced invaginations of the epithelium’s basal lamina at the contact sites between the PPG and these neighboring cells as well as at sites where the PPG is in direct contact to the hemocoel suggest an extensive transport of substances across this basal lamina. 

### Chemistry of *Trachypus* PPG and cuticle

In the PPG samples of *T. boharti*, we detected 51 substances with chain lengths of 21-31 carbon atoms (Table 1). The chemical profile was dominated by long-chain unsaturated HCs (on average 84.5±1.5% of the total amount of HCs) with 7-pentacosene being the most abundant compound. The PPGs of *T. boharti* also contained 14 long-chain unsaturated ketones ranging from C23 to C29. The structures of these alkenones were assigned by their mass spectra and the mass spectra of their DMDS adducts. The mass spectrum of the most abundant alkenone, 18-pentacosen-6-one, for example, was characterized by a molecular ion at *m/z* 364 [C5H11-CO-C19H37]^+^ and two diagnostic fragment ions at *m/z* 99 for [C_5_H_11_-CO]^+^ and at *m/z* 293 for [C_19_H_37_-CO]^+^, indicating that the carbonyl group is at position C6. The mass spectrum of the DMDS (molecular weight = 94) adduct showed a molecular ion at m/z 458 (364+94) and diagnostic fragment ions at *m/z* 145 and *m/z* 313, which clearly reflect a double bond at the C18 position in the original alkenone (see Table 1 and Figure S1 for the diagnostic ions of the ketones and their respective DMDS adducts and two exemplary mass spectra). The mass spectrum of 18-heptacosen-10-one was identical to that published by Yasui et al. [43]. 14-tricosen-6-one, 16-pentacosen-8-one, 18-heptacosen-10-one had previously been described for *P. triangulum* [17]. Mass spectra and retention times of these ketones found in *T. boharti* and *P. triangulum* (reanalyzed on our GC/MS set-up) were identical. 

**Table 1:** Chemical composition of the postpharyngeal gland content and the cuticle of *T. boharti* females.

**No.**	**Substance**	**LRI**	**Presence on Cuticle**	**Diagnostic ions**
B1	Heneicosane	2100	+	296
B2	9-Tricosene	2272	+	322; DMDS: 173, 243, 416
B3	7-Tricosene	2280	+	322; DMDS: 145, 271, 416
B4	Tricosane	2300	+	324
B5	9-Tetracosene	2372	+	336; DMDS: 173, 257, 430
B6	7-Tetracosene	2380	+	336; DMDS: 145, 285, 430
B7	Tetracosane	2400	+	338
B8	14-Tricosen-6-one	2453	-	99, 181, 265, 336; DMDS: 99, 173, 257, 430
B9	14-Tricosen-4-one	2461	-	71, 153, 293, 336; DMDS: 71, 173, 257, 430
B10	+ 16-Tricosen-6-one		-	99, 181, 265, 336; DMDS: 99, 145, 285, 430
B11	16-Tricosen-4-one	2467	-	71, 153, 293, 336; DMDS: 71, 145, 285, 430
B12	+ Pentacosadiene		-	348; DMDS: n.d.
B13	+ 3-Methyltetracosane		-	57, 323
B14	9-Pentacosene	2473	+	350; DMDS: 173, 271, 444
B15	7-Pentacosene	2484	+	350; DMDS: 145, 299, 444
B16	5-Pentacosene	2491	+	350; DMDS: 117, 327, 444
B17	Pentacosane	2500	+	352
B18	13-Methylpentacosane	2533	+	196/197 (sym.)
B19	+ 11-Methylpentacosane		+	168/169, 224/225
B20	5-Methylpentacosane	2548	-	85, 308/309
B21	9-Hexacosene	2573	+	364; DMDS: 173, 285, 458
B22	8-Hexacosene	2577	-	364; DMDS: 159, 299, 458
B23	7-Hexacosene	2582	+	364; DMDS: 145, 313, 458
B24	Hexacosane	2600	+	366
B25	16-Pentacosen-8-one	2654	+	127, 209, 265, 364; DMDS: 127, 173, 285, 458
B26	16-Pentacosen-6-one	2659	+	99, 181, 293, 364; DMDS: 99, 173, 285, 458
B27	18-Pentacosen-8-one	2663	+	127, 209, 265, 364; DMDS: 127, 145, 313, 458
B28	18-Pentacosen-6-one	2666	+	99, 181, 293, 364; DMDS: 99, 145, 313, 458
B29	6,9-Heptacosadiene	2670	+	376; DMDS: 131, 155, 203, 299, 323, 371, 407, 455, 502
B30	9-Heptacosene	2675	+	378; DMDS: 173, 299, 472
B31	3, 6, 9-Heptacosatriene	2679	+	108, 135, 318, 345, 331, 374
B32	7-Heptacosene	2684	+	378; DMDS: 145, 327, 472
B33	5-Heptacosene	2693	+	378; DMDS: 117, 355, 472
B34	Heptacosane	2700	+	380
B35	7-Octacosene	2784	+	392; DMDS: 145, 341, 486
B36	18-Heptacosen-10-one	2860	-	155, 237, 265, 392; DMDS: 155, 173, 313, 486
B37	+ 18-Heptacosen-8-one		-	127, 209, 293, 392; DMDS: 127, 173, 313, 486
B38	20-Heptacosen-10-one	2864	+	155, 237, 265, 392; DMDS: 155, 145, 341, 486
B39	+ 20-Heptacosen-8-one	2867	+	127, 209, 293, 392; DMDS: 127, 145, 341, 486
B40	6,9-Nonacosadiene	2873	+	404; DMDS: 131, 155, 203, 327, 351, 399, 435, 483, 530
B41	9-Nonacosene	2876	+	406; DMDS: 173, 327, 500
B42	3, 6, 9-Nonacosatriene	2881	+	108, 135, 346, 359, 373, 402
B43	7-Nonacosene	2884	+	406; DMDS: 145, 355, 500
B44	Nonacosane	2900	+	408
B45	7-Triacontene	2986	+	420; DMDS: 145, 369, 514
B46	22-Nonacosen-12-one	3069	-	183, 265, 420; DMDS: 171, 145, 369, 514
B47	+ 22-Nonacosen-10-one		-	155, 237, 293, 420; DMDS: 155, 145, 369, 514
B48	Hentriacontadiene	3077	+	432; DMDS: n.d.
B49	+ 9-Hentriacontene		+	434; DMDS: 173, 355, 528
B50	7-Hentriacontene	3086	+	434; DMDS: 145, 383, 528
B51	Hentriacontane	3100	+	436

LRI = linear retention index (calculated in relation to *n*-alkanes) on the RH-5ms+ column;

^+^ = present;

^-^ = absent;

sym = symmetric molecule, reduced number of diagnostic ions; DMDS = diagnostic ions of the respective DMDS adducts;n.d. = not detected.

The polar as well as non-polar fractions of pooled samples revealed the presence of additional compounds (mainly saturated and unsaturated ketones) which could not be detected in extracts from single individuals because of their low abundance and/or because they coeluted with the dominant HCs. For completeness, these additional compounds are given in Table S1. 

The cuticle samples of *T. boharti* contained 39 substances of chain length C21 to C31 (Table 1). The overall chromatographic patterns of PPG and cuticle closely resembled each other. According to a major axis regression analysis (R=0.88, P=0.0003, see Figure S2), there was a direct proportionality in the proportions of components on the cuticle and in the PPG.

The average total amount of substances found in the PPG samples of *T. boharti* was 15.6 ± 6.4 µg, the average total amount of compounds on the cuticle was 8.2 ±3.1 µg and thus significantly less than in the PPG (paired t-test: t=-3.99, P=0.0032). 

**Figure 5:**
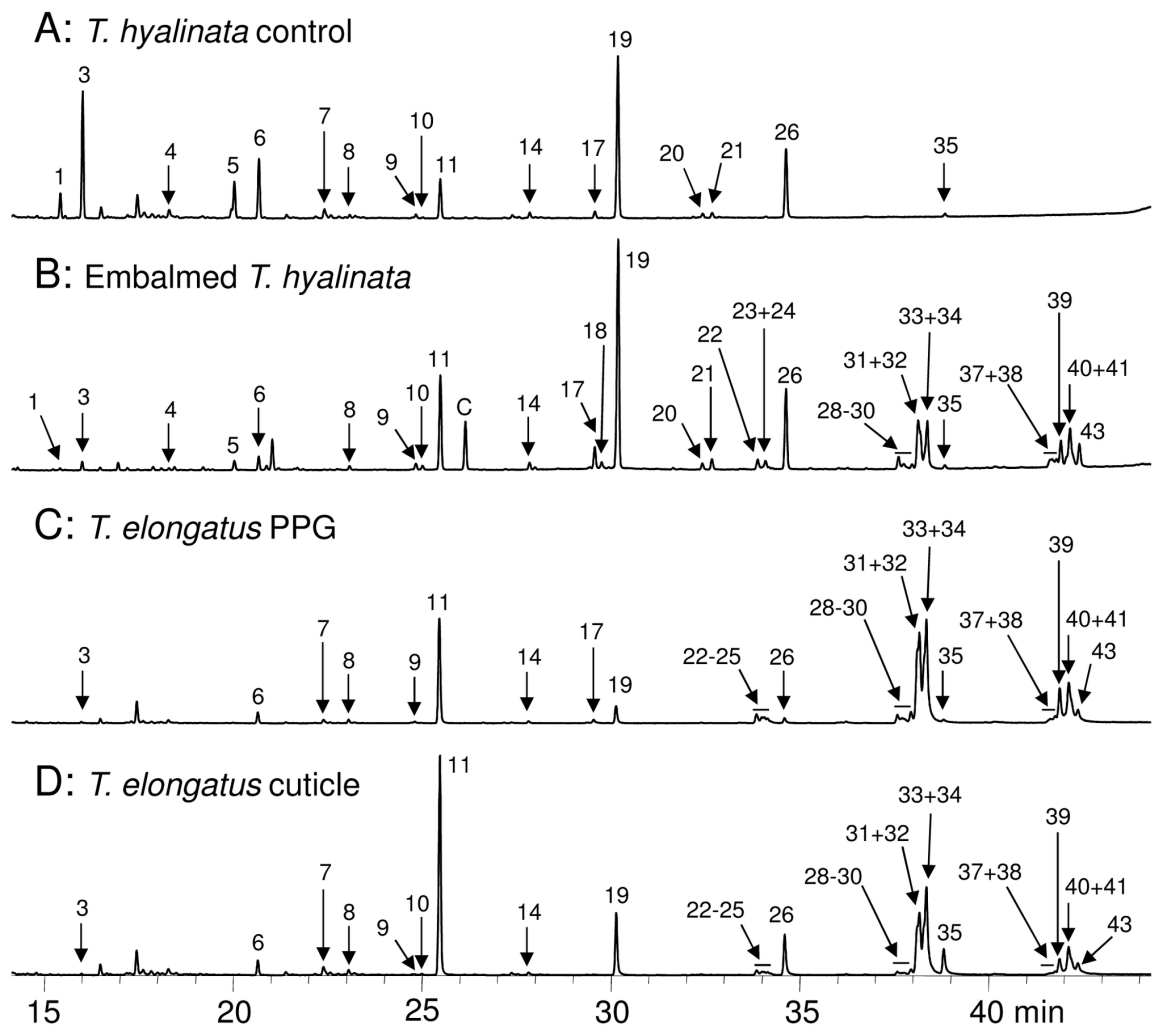
Chromatographic evidence for prey embalming by *T. elongatus* females. Total ion chromatograms of (**A**) a *T. hyalinata* control bee, (**B**) a provisioned *T. hyalinata* bee excavated from a *T. elongatus* nest, (**C**) the PPG content of a *T. elongatus* female and (**D**) the cuticle of the same *T. elongatus* female. The peaks of minor compounds are not always visible due to the magnification used. The numbers at the peaks correspond to the numbers in Table 2. Unlabelled peaks are contaminations.

In *T. elongatus*, the PPG and head samples contained 35 substances with chain length C21 to C33 (Table 2 and Figure 5C) and were dominated by unsaturated HCs (67%). The cuticle samples contained the same substances, and the chromatographic pattern of the PPG showed high congruence with the cuticular chemistry (R=0.88, P=0.004, Figures 5C and D and Figure S3). The total amount of HCs was 47.5 µg for the PPG and 54.1µg for the corresponding cuticle sample. 

**Table 2:** Comparison of the chemistry of *T. elongatus* with its provisioned prey and control bees.

**No.**	**Substance**	**LRI**	**PPG**	**Cuticle**	**EB**	**CB**	**Diagnostic ions**
1	Heneicosene	2072	-	-	+	+	294
2	Heneicosene	2078	-	-	-	+	294
3	Heneicosane	2100	+	+	+	+	296
4	Docosane	2200	+	+	+	+	310
5	Tricosene	2273	-	-	+	+	322
6	Tricosane	2300	+	+	+	+	324
7	3-Methyltricosane	2372	+	+	+	+	85, 309
8	Tetracosane	2400	+	+	+	+	338
9	9-Pentacosene	2473	+	+	+	+	350; MS2: 306
10	7-Pentacosene	2479	+	+	+	+	350; MS2: 334
11	Pentacosane	2500	+	+	+	+	352
12	5-Methylpentacosane	2549	+	+	+	-	85, 308/309
13	3-Methylpentacosane	2573	+	+	+	+	57, 337
14	Hexacosane	2600	+	+	+	+	366
15	13-Heptacosene	2666	+	-	-	-	378; MS2: 278
16	11-Heptacosene	2669	+	-	-	-	378; MS2: 306
17	9-Heptacosene	2675	+	+	+	+	378; MS2: 334
18	Heptacosene	2684	-	-	+	+	378
19	Heptacosane	2700	+	+	+	+	380
20	Octacosane	2800	-	-	+	+	394
21	Unidentified	2811	-	-	+	+	-
22	13-Nonacosene	2866	+	+	+	-	406; MS2: 306
23	10-Nonacosene	2873	+	+	+	-	406; MS2: 348
24	9-Nonacosene	2876	+	+	+	+	406; MS2: 362
25	8-Nonacosene	2879	+	+	+	-	406; MS2: 376
26	Nonacosane	2900	+	+	+	+	408
27	Triacontane	3000	-	-	+	-	422
28	9,19-Hentriacontadiene	3041	+	+	+	-	432; MS2: 374, 332
29	Hentriacontadiene	3048	+	+	+	-	432
30	Hentriacontadiene	3058	+	+	+	-	432
31	15-Hentriacontene	3066	+	+	+	-	434; MS2: 306
32	12-Hentriacontene	3069	+	+	+	-	434; MS2: 348
33	10-Hentriacontene	3075	+	+	+	-	434; MS2: 376
34	9-Hentriacontene	3078	+	+	+	-	434; MS2: 390
35	Hentriacontane	3100	+	+	+	+	436
36	Dotriacontane	3200	-	-	+	-	450
37	8,22-Tritriacontadiene	3241	+	+	+	-	460; MS2: 416, 374
38	Tritriacontadiene	3250	+	+	+	-	460
39	Tritriacontadiene	3258	+	+	+	-	460
40	16-Tritriacontene	3262	+	+	+	-	462; MS2: 320
41	14-Tritriacontene	3264	+	+	+	-	462; MS2: 348
42	12-Tritriacontene	3271	+	+	+	-	462; MS2: 376
43	9-Tritriacontene	3277	+	+	+	-	462; MS2: 418

Chemical composition of solvent extracts of the postpharyngeal gland (**PPG**) and cuticle (**Cuticle**) of a *T. elongatus* female, a *T. hyalinata* prey bee excavated from a *T. elongatus* nest (**EB**, embalmed bee) and a *T. hyalinata* control bee (**CB**, control bee).

LRI = linear retention index (calculated in relation to *n*-alkanes) on the RH-5ms+ column;

^+^ = the substance was detected in at least one of the samples;

^-^ = not detected;

sym = symmetric molecule, reduced number of diagnostic ions;

MS2 = MS2 diagnostic ions (MS2 precursor ion for all compounds: [M+54]**^*+*^**).

### Prey embalming in *T. elongatus*

Of the 31 excavated *T. elongatus* brood cells, three were freshly provisioned and contained 4, 3, and 3 *Trigona hyalinata* (Lepeletier) (Hymenoptera, Apidae, Meliponini) workers, respectively. Chemical analyses showed that *T. hyalinata* control bees that were caught in the vicinity of *T. elongatus* nests and directly from their nest entrance carried 21 HCs (Figure 5A and Table 2). The chemical analyses of the provisioned bees revealed that, in addition to the species specific cuticular HCs, they all carried the substances that we found in the samples of *T. elongatus* females (Figure 5B and Table 2). 

**Figure 6:**
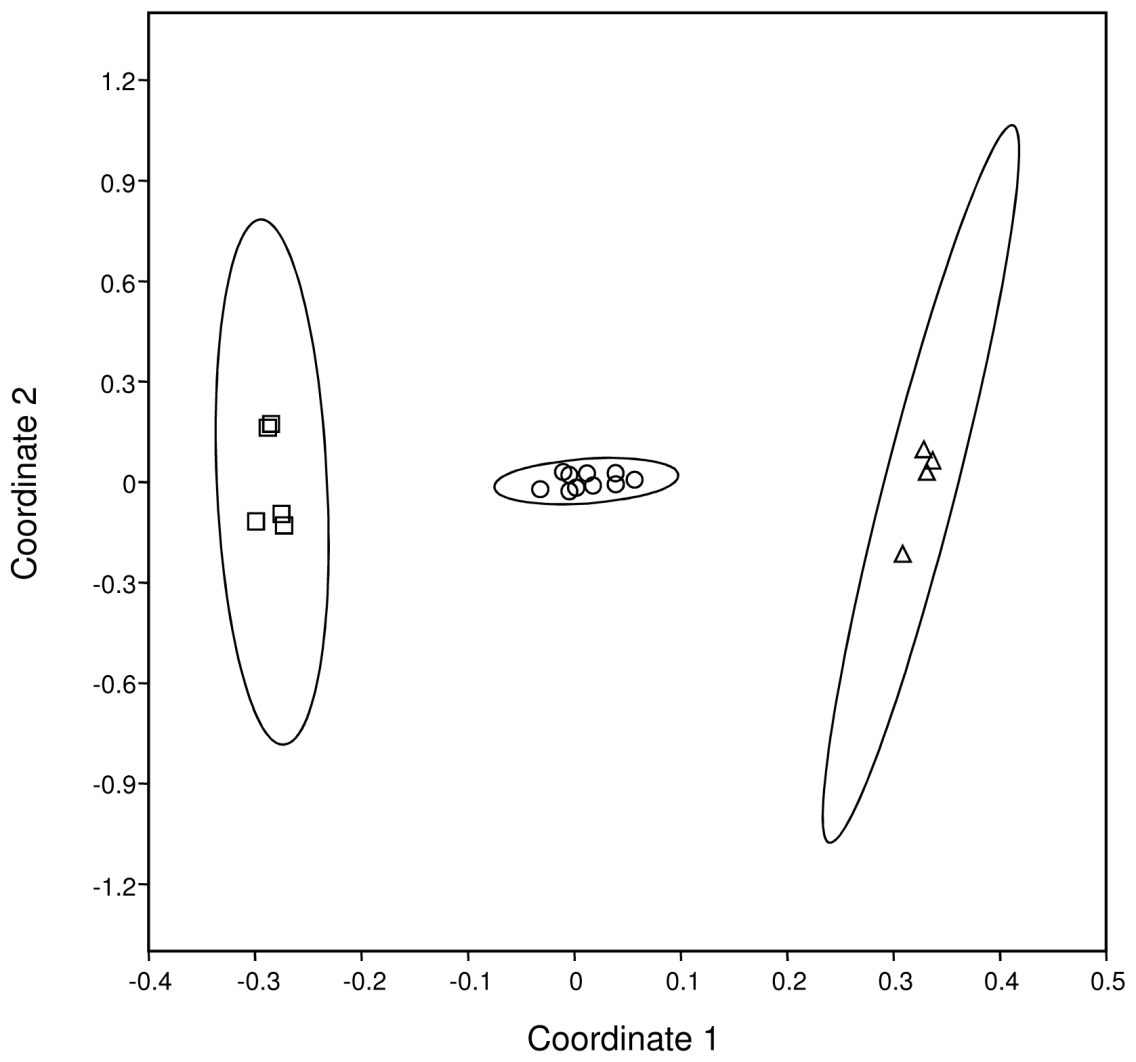
Statistical evidence for prey embalming by *T. elongatus* females. Two-dimensional MDS representation of the chemical profiles of individual *T. hyalinata* control bees (squares), provisioned *T. hyalinata* bees (circles), and *T. elongatus* females (triangles) (stress value: 0.11). The ellipses depict the 95% confidence intervals.

If *T. elongatus* females add their characteristic HCs to the surface of their prey bees that already carry their own characteristic HCs, the chemical profile of provisioned bees is expected to be intermediate between the wasps and conspecific control bees. The multivariate statistical analyses revealed that this was actually the case (Figure 6). An ANOSIM analysis showed an overall significant difference between all three groups (R = 0.97, P = 0.0001) and significant differences in all pairwise comparisons (sequential Bonferroni correction; P < 0.01 for all comparisons; control bees vs. provisioned bees R = 0.96; control bees vs. *T. elongatus* R = 1; provisioned bees vs. *T. elongatus* R = 0.99). The substances contributing most to the difference between control bees and provisioned bees were the hentriacontenes (15% in the SIMPER analysis), which are the most abundant unsaturated compounds found in the PPG of *T. elongatus* females (see also Figure 5 and Table 2).

Provisioned bees carried only slightly but not significantly more HCs than control bees (median ± median absolute deviation: 40.9 ± 2.2 µg *vs.* 38.7 ± 4.9 µg; exact P=0.77). Nonetheless, the proportion of unsaturated HCs was significantly higher on provisioned bees as compared to the controls (median ± median absolute deviation = 37.3 ± 7.0% *vs*. 18.3 ± 0.30%; exact P=0.0013). 

## Discussion

### Morphology and ultrastructure of *Trachypus* PPGs

Females of *T. boharti* and *T. elongatus* possess large PPGs. The location and basic morphology are similar to the PPGs of female European beewolves, *P. triangulum* [16], as well as many ant species [19-21]. There are, however, some notable differences. While in *T. boharti* and *P. triangulum* the PPG consists of a pair of evaginations, each with its own connection to the pharynx, the two branches of the PPG of *T. elongatus* cohere at their base and possess only one joint connection to the pharynx. The lower, smaller part of the PPG with a separate opening to the pharynx that has been described for *P. triangulum* does not occur in the two *Trachypus* species. The more or less pronounced extension of the PPG ventrally to the pharynx in *T. elongatus* might be interpreted as an intermediate stage between *P. triangulum* and *T. boharti*. The estimated gland volume of *P. triangulum* is about ten times larger than that of *T. elongatus*. The gland volume of *T. boharti* was even smaller. However, the gland tubes of *T. boharti* appeared partly collapsed, so the actual gland volume is probably somewhat larger. 

As in *P. triangulum*, the wall of the PPG in *Trachypus* is a monolayered epithelium, and the lumen of the gland is lined by a cuticular intima. The PPG thus belongs to the epithelial type. The long hairs that are formed by the cuticular intima and extend far into the lumen have also been described for the PPG of *P. triangulum*. Also as in *P. triangulum*, the PPG of *Trachypus* is partly bordered by two different types of cells and there are no muscles associated with the epithelium of the gland.

The ultrastructural investigations of *T. boharti* revealed that the cells bordering the epithelium of the PPG show a considerable density of smooth and rough endoplasmatic reticulum and thus appear to be synthetically very active. The cells of the PPG epithelium show comparatively few signs of synthetic activity. The pronounced invaginations of the basal lamina at the contact areas between the PPG and the neighboring cells as well as the hemolymph rather suggest that there is an intense transport of substances across the epithelium of the PPG. We therefore propose that the secretion that is stored in the PPG of *Trachypus* females is at least partly sequestered from the hemolymph and might be synthesized in the neighboring cells and in the fat body. Such a (at least partial) sequestration of compounds into the PPG has also been proposed for female *P. triangulum* [16,18] and the emerald cockroach wasp *A. compressa* [23] and has been shown in ants (see below) [20,44]. 

### Chemistry of *Trachypus* PPG and cuticle

As in *P. triangulum* [17,18], *A. compressa* [23] and various ant species [20,25], the PPGs of *T. boharti* and *T. elongatus* contain mostly HCs. The chemistry of the two *Trachypus* species is dominated by long-chain unsaturated HCs, which is also true for *P. triangulum* [17]. The PPG of *T. elongatus* contains more doubly-unsaturated HCs than that of *T. boharti* and *P. triangulum*. In both *T. boharti* and *P. triangulum* the by far most prominent compound is pentacosene, while in *T. elongatus* the hentriacontenes and pentacosane are the most abundant compounds. Interestingly, the PPG of *T. boharti* additionally comprises long-chain ketones. 14-tricosen-6-one, pentacosan-8-one, heptacosan-10-one, 16-pentacosen-8-one, and 18-heptacosen-10-one have also been found in the PPG, on the cuticle and in the hemolymph of *P. triangulum* [17]. However, we did not find any ketones in *T. elongatus*. 18-heptacosen-10-one and heptacosan-10-one are part of the contact sex pheromone in the white-spotted longicorn beetle, *Anoplophora malasiaca* [43]. All other unsaturated ketones have, to our knowledge, not yet been described as natural products. Whether these ketones play a special role for the defense against microbes and why *T. elongatus* lacks these ketones is not yet known.

In ants, there is convincing evidence that the HCs are synthesized by oenocytes, released into the hemolymph, transported by lipophorin to the cuticle as well as to the PPG and continuously exchanged between these two sites [20,44]. Consequently, there is congruence of the HC profiles of the hemolymph, the PPG and the cuticle [20,25,45]. Such congruence was also shown in *P. triangulum* [18] and *A. compressa* [23]. In the two *Trachypus* species, we also found a high congruence between the chemical profiles of PPG and cuticle, suggesting a similar mode of synthesis and circulation of HCs.

The amount of HCs in the *T. elongatus* PPG was 48 µg. The PPGs of field caught *T. boharti* contained on average 15.6 µg HCs and varied considerably between 4.8 µg and 27.4 µg. The average amount of HCs found in the PPG of field caught *P. triangulum* females (330µg) was considerably higher [16]. This difference is only partly explained by the larger size of *P. triangulum* females (average head capsule width 4.25-4.5mm [46] as opposed to *T. boharti* (3.4±0.2mm; N=16) and *T. elongatus* females (3.6±0.1mm; N=3), making up for an approximately twofold difference in body volume. Thus, the comparatively smaller glands and lower amounts of secretion in the two *Trachypus* species under study must have different reasons (see below).

### Prey embalming

The specific HCs from the PPG of *T. elongatus* were clearly present on provisioned *T. hyalinata* bees excavated from *T. elongatus* nests, but not on *T. hyalinata* control bees. Our chemical and statistical analyses revealed that *T. elongatus* females add enough long-chain unsaturated HCs from their PPGs to their prey bees to significantly change their chemical profile. Consequently, the surface chemistry of provisioned *T. hyalinata* bees was intermediate to the chemical profiles of *T. hyalinata* control bees and *T. elongatus* females. For the present study provisioned prey of *T. boharti* was not available. It is tempting to speculate, however, that this species, which preys exclusively on males of *Scaptotrigona postica* (Latreille) (Hymenoptera, Apidae, Meliponini) [32,33] embalms its prey with the secretion of its PPG.

In *T. elongatus* the embalming did not significantly elevate the total amount of HCs on the bees’ surface. This is in contrast to *P. triangulum*, where embalming results in a five to tenfold increase of HCs on the prey surface [10,11]. However, the quantity of secretion applied to the bees might not be the decisive factor for the adaptive significance of prey embalming in *Trachypus*. In *P. triangulum* the embalming not only changes the quantity but also the quality of HCs on the bees by increasing the proportion of unsaturated HCs about threefold (25.8±7.8% for control bees and 73.6±9.9% for provisioned bees; [10]). This alters the physicochemical properties of the bee surface and consequently causes a decrease in water condensation. The reduced availability of water on the bees in turn renders microclimatic conditions unfavorable for the growth of competing mold fungi. In *P. triangulum* already small amounts of HCs on the bees were sufficient to mediate this physical effect [10], indicating that the quality of secretion, namely its high degree of unsaturation, is probably more important than its quantity (see 13 for a more detailed discussion).

As in *P. triangulum*, the PPG secretions of both *Trachypus* species consist primarily of unsaturated long-chain HCs. Consequently, in *T. elongatus* the embalming of the provisioned bees with the PPG secretion significantly elevated the proportion of unsaturated compounds on their surface as compared to not embalmed control bees. Whether this doubling of the proportion of unsaturated HCs on the surface of the prey of *T. elongatus* is sufficient to mediate the effect of reduced water condensation and fungus growth is currently unclear. Assuming the same relationship between the proportion of unsaturated HCs and water gain as has been found in *P. triangulum* in the laboratory [10], an increase of unsaturation from 18 to 37% would cause a two-fold reduction in water condensation on *T. elongatus* prey bees. Furthermore, the excavated *T. elongatus* brood cells were found very deep in the soil (at 95, 100, and 115 cm below the surface), where temperature fluctuations that would favor excessive water condensation are expected to be negligible [47]. Together, these effects might be sufficient to effectively impair fungal growth. 

The second benefit of prey embalming in *P. triangulum*, the reduction of evaporative water loss of the prey, increases with increasing amounts of HCs on the prey surface [12]. As the brood cells of *T. elongatus* were located deep in the soil and surrounded by rather moist sand, the desiccation of the prey might not be a problem for *T. elongatus*. Thus, also the second function of the embalming, the prevention of desiccation, would not select for large amounts of HCs as a water barrier as found in *P. triangulum*. 

In addition to the benefits of prey embalming, the accompanying costs have to be considered. In *P. triangulum*, prey embalming has been shown to enhance offspring survival but also to entail costs with regard to a reduced future reproductive potential of the females [13]. In order to maximize their fitness returns, female *P. triangulum* hence might adjust the amount of secretion in response to the actual conditions. In general, the optimal amount of secretion applied to the prey bees might depend on various biotic and abiotic factors, i.e. the nesting site, temperature and humidity, the prey species, the number of prey items and the threat by pathogenic and competing microorganisms. The prevalent prey of *Trachypus*, stingless bees, are known to carry plant-derived resins on their cuticles [48,49], and these mostly terpenoid compounds are known to have antimicrobial activity [50]. In the present study we found indications that *T. hyalinata* likewise carries resins on its surface (As the incidence of these compounds was irregular and their analysis was beyond the scope of the study, we do not provide more details.). Such resins might decrease the susceptibility of *T. hyalinata* to mold fungi and *Trachypus* females might therefore invest less in prey embalming. 

As most of the abiotic and biotic factors that may shape prey preservation are likely to differ among *Philanthus* and *Trachypus* species, each species can be expected to adjust to the level of embalming that maximizes its overall reproductive success over evolutionary times. It is therefore conceivable that the optimal embalming strategy may differ between the European beewolf *P. triangulum* and the South American beewolf *T. elongatus*. We thus propose that, despite the lower amounts of secretion applied, the prey embalming in *Trachypus* has evolved as a parental care strategy for the purpose of prey preservation.

## Conclusions

The South American beewolves of the genus *Trachypus*, which show a very similar life-history to the closely related genus *Philanthus*, seem to deploy similar antimicrobial defense mechanisms like the protective symbiosis with bacteria and the embalming of the prey with long-chain unsaturated HCs. Despite the similarities in morphology and chemistry of the PPG as well as the prey embalming between *P. triangulum* and the two *Trachypus* species under study, our results show that there are also considerable differences. These are probably the result of diverging selection pressures with regard to biotic and abiotic conditions that have shaped these traits in the different species over evolutionary times. Further studies on the genera *Philanthus* and *Trachypus*, other members of the subfamiliy Philanthinae, and even more distantly related digger wasps will be necessary to further unravel the origin and evolutionary history of the PPG and the exceptional antifungal defense mechanism of prey embalming. 

## Methods

### Ethics statement

Permits were issued by the Brazilian Ministry of the Environment: MMA/SISBIO/22861-1.

### Specimens and nest excavations

Adult female *Trachypus boharti* and *T. elongatus* were collected in São Paulo State, Brazil. Of the collected *T. boharti*, five heads were fixed for histological investigations; six heads were dissected to remove the PPGs for ultrastructural investigations. Ten specimens were dissected to remove the PPGs for chemical analyses. The corresponding bodies (thorax and abdomen) were used for chemical analyses. A total of five*T. elongatus* females was collected. Three heads were fixed for histological investigations, one head was extracted in hexane for chemical analysis, and one head was dissected to remove the PPG for chemical analysis. Of the corresponding bodies four were extracted in hexane for chemical analyses. Nests of still active *T. elongatus* females were excavated to obtain prey bees from freshly provisioned brood cells. For comparison, individuals of the prey species of *T. elongatus*, the stingless bee *Trigona hyalinata*, were caught directly from their nest entrance. 

### Histological investigations

Histological investigations of *T. boharti* (N = 5) and *T. elongatus* (N = 3) heads were conducted using light microscopy following standard histological methods. Freshly caught *Trachypus* were cold-anaesthetized and decapitated. The heads were fixed immediately in formalin-ethanol-acetic acid fixative (Scheuring). Subsequently, they were rinsed in 80% ethanol and both eyes were cut off using razor blades to enable the embedding medium to soak into the head. The specimens were then dehydrated in a graded ethanol series and propylene oxide, and finally embedded in Epon 812 (Polysciences Europe GmbH, Eppelheim, Germany). Continuous series of semi-thin sections (4 µm) were cut on a microtome (Reichert Ultracut; Leica Microsystems AG, Wetzlar, Germany) equipped with a diamond knife and stained with toluidine blue [51]. Sections were examined under a compound microscope (Zeiss Axiophot) and photographs were taken using a Nikon digital camera (Nikon Digital Sight DS-2Mv) and Nikon NIS F 2.20 software (Nikon Corp., Tokyo, Japan).

### 3D-reconstruction

3D-reconstructions of one *T. boharti* and one *T. elongatus* female head based on the series of semi-thin sections were conducted using the 3D visualization software Reconstruct (SynapseWeb, Kristen M. Harris, PI, http://synapses.clm.utexas.edu/). The photographs of the series of head sections were loaded into the program and manually aligned. The structures of interest (postpharyngeal gland, pharynx, brain, ocelli) were manually marked in each picture and the 3D surfaces computed. The gland volumes were calculated by the Reconstruct software. 

### Transmission electron microscopy

Six *T. boharti* females were cold anaesthetized, decapitated and their heads dissected under a stereomicroscope to remove the PPGs as described previously for *P. triangulum* [22]. The PPGs were then fixed for transmission electron microscopy (TEM) in a modified Karnovsky’s prefixative solution (2.5% glutardialdehyde and 2 % paraformaldehyde in sodium dihydrogen orthophosphate buffer; pH 7.4) in a refrigerator at approximately 4 °C. After postfixation in 2% OsO4 in 0.1M sodium dihydrogen orthophosphate buffer (pH 7.4) on ice for 2 hrs, the specimens were dehydrated in a graded ethanol series. They were then embedded in Epon 812 (with acetone as the intermediary solution). Ultra-thin sections were made with a 45° diamond knife on a Reichert Ultracut E microtome (Leica Microsystems AG, Wetzlar, Germany). Sections were stained with 2% uranyl acetate and Reynolds’ lead citrate and examined with a Zeiss EM 10 at 80 kV. Micrographs were taken with a TRS slow scan CCD camera (type s-7899-v) and the software TRS Image Sys Prog (Version 1.1.1.65).

### Chemical analyses

The solvent extracts of the cuticles and the PPGs of *T. boharti* and *T. elongatus* were analyzed by gas chromatography / mass spectrometry (GC/MS, see below) to identify and quantify the chemical compounds and to test for a possible chemical congruence. The PPGs were obtained as described above and transferred to hexane vials (one PPG per vial), where they remained until chemical analyses were conducted. The respective bodies (thoraces and abdomens) as well as the one head of *T. elongatus* were individually extracted in hexane for 10 min. The bees excavated from *T. elongatus* nests (N = 10) as well as control bees (N = 5) were individually extracted in hexane for 10 min. Octadecane was added as an internal standard for quantification of the HCs, the samples were reduced in volume to approximately 70µl under a gentle stream of nitrogen and 1µl of each sample was analyzed by GC/MS. 

GC/MS analysis was performed with an Agilent 6890N Series gas chromatograph coupled to an Agilent 5973 inert mass selective detector (Agilent Technologies, Böblingen, Germany). The GC was fitted with an RH-5ms+ fused silica capillary column (30m×0.25mm ID; film thickness = 0.25µm; Capital Analytical Ltd., Leeds, England). The GC was programmed from 70 to 180 °C at 30 °C/min and then at 3°C/min to 300 °C, with a 1-min initial isothermal and a 5-min final isothermal hold. A split/splitless injector (250 °C) was used in the splitless mode, with the purge valve opened after 1min. Helium was the carrier gas at a constant flow rate of 1ml/min. Electron ionization mass spectra (EI-MS) were recorded at an ionization voltage of 70 eV, a source temperature of 230 °C, and an interface temperature of 315 °C. Data acquisition and storage were performed with the GC/MS software MSD ChemStation for Windows (Agilent Technologies, Palo Alto, CA, USA). Peak areas were obtained by manual integration using the GC software.

After they had been run individually on the GC/MS, the crude extracts of *T. boharti* were pooled and then fractionated by column chromatography on a silica gel column (Chromabond 100mg, Macherey and Nagel) with hexane and dichloromethane as the mobile phase to separate polar from non-polar compounds. An aliquot of 1µl of each fraction was analyzed by GC/MS. Hydrocarbons were identified as described previously [23]. Iodine-catalyzed methylthiolation using dimethyl disulfide (DMDS) [52,53] was performed to determine the positions of the double bonds in alkenes, alkadienes and alkenons. Double-bond positions in the alkatrienes were determined by their characteristic fragments, which, together with the molecular ion, allow the assignment of double bonds [54]. Details on the identification of polar compounds are given in the results section. 

For *T. elongatus*, double bond positions of unsaturated HCs were determined by a chemical ionization (CI) tandem-mass spectrometry (MS/MS) *in situ* double bond derivatization method using acetonitrile (ACN) as the reagent gas [55]. As this technique does not result in a retention time shift of the derivatized compounds, double bond positions can also be determined for very long-chain HCs that are not amenable to DMDS derivatization. ACN-CI-MS/MS was performed using a Varian 240MS ion-trap mass detector coupled to a Varian 450GC. All analyses were done in the internal ionization configuration. A DB-WAXETR column (30m×0.25mm ID, 0.25μm df; Agilent Technologies, Santa Clara, CA, USA) was used for optimal separation of HC isomers. The split/splitless injector was operated at 250°C in the splitless mode. The GC oven temperature was programmed as follows: 80°C for 2min, 15°C/min to 240°C, hold 30min. Helium was used as the carrier gas at a constant flow rate of 1ml/min. CI spectra were recorded using acetonitrile (ACN) as the reagent gas with a mass range of m/z 60-500. CI-MS/MS experiments with the [M+54]^+^ adducts of unsaturated HCs as precursor ions were conducted with the resonant waveform type for precursor ion excitation, an isolation window of m/z = 3, and a maximum reaction time of 100ms. The automatic “q” calculator was used for the determination of precursor ion excitation energy and product ion mass range. All CI measurements were conducted with an ion-trap temperature of 220°C, a manifold temperature of 50°C, and a transfer line temperature of 240°C. 

Linear retention indices (LRIs) of unsaturated HCs were determined on a DB-5ms column (30m×0.25mm ID, 0.25μm df; Agilent Technologies, Santa Clara, CA, USA) with the following GC oven temperatures: 150°C for 1min, 5°C/min to 300°C, hold 10min. All other settings were identical to the ones used for identification of HCs on the DB-WAXETR column. EI-MS and ACN-CI-MS/MS experiments were used to homologize peaks with those detected on the DB-WAXETR column. Data were analyzed using the MS Workstation software (version 6.9.3, Varian Inc., Palo Alto, CA, USA).

### Data analysis

To test for a chemical congruency between the HCs found in the PPG and on the cuticle of *T. boharti*, a major axis regression analysis between the mean proportions of components in the PPG and on the cuticle was conducted (for further details see Figure S2). The total amounts of substances in the individual samples were calculated by use of the internal standard. For *T. boharti* the mean amounts in the PPG and on the cuticle were compared with a paired t-test. If not otherwise stated, data given are means ± standard deviation.

For *T. elongatus* females, we obtained chemical data of the PPG and cuticle of one individual, the head and cuticle of another individual and the cuticles of two further individuals. We checked for a chemical congruence of the PPG, head and cuticle samples by investigation of the chromatographic patterns, by regression analysis and by multivariate statistical analyses (see Figure S3 and Figure S4). The chromatographic patterns as well as the statistical analyses revealed 1) that the chemical profiles of the four individuals were very similar and 2) that there was a strong linear relationship between the PPG/head samples and the corresponding cuticle samples, as has been shown for *T. boharti* in the present study (see Results) as well as for the closely related *P. triangulum* [18] and the more distantly related *A. compressa* [23] in earlier studies. The chemical congruence of PPG and cuticle justifies the inclusion of the cuticle samples in our statistical analyses that test the hypothesis of prey embalming. All further statistical analysis were performed with the dataset containing one sample per *T. elongatus* individual (N=4 cuticle samples).

To assess whether *T. elongatus* females embalm their prey, we compared the chemical profiles of *T. hyalinata* control bees (N=5), the excavated provisioned *T. hyalinata* bees (N=10) and *T. elongatus* females (N=4) by investigation of the chromatographic patterns as well as by multivariate statistical analyses. As the different isomers of the alkadienes and alkenes were only partially resolved by chromatography, they were integrated together, which resulted in a total of 22 integrated peaks. The total peak area of each individual extract was standardized to 100% and the relative peak areas were calculated. We visualized differences in the chemical profiles between the groups by non-metric multidimensional scaling (nmMDS), based on Bray-Curtis similarity measures [56,57]. The significance of the differences between groups was assessed by one-way ANOSIM (ANalysis Of SIMilarity) and the chemical compounds primarily responsible for the observed differences between control and provisioned bees were identified using a SIMPER (SIMilarity PERcentage) analysis, both based on Bray-Curtis similarity indices.

The total amount of HCs as well as the proportion of unsaturated HCs on provisioned and control bees was calculated and compared with exact tests for independent samples. All statistical analyses were performed with the statistics software package PAST (Version 2.15, [58]).

## Supporting Information

Figure S1 **Mass spectra for (**A**) 18-pentacosen-6-one and (**B**) its DMDS adduct.**
(TIF)

Figure S2 **Congruence between PPG content and cuticle in *T. boharti*.** The overall chromatographic patterns of PPG and cuticle closely resembled each other. All substances found on the cuticle were also present in the PPG. Twelve compounds were only detected in the PPG but not on the cuticle. As these substances are all minor compounds and the cuticle samples in general contained lower amounts of compounds, the twelve compounds were most probably below the detection limit in the cuticle samples. A major axis regression between the mean relative amounts of eleven selected peaks (representing 17 compounds) revealed a strong linear relationship between the compounds in the gland and the corresponding compounds on the cuticle (R=0.88, P=0.0003, N=10 individuals). Moreover, the linear regression was consistent with a direct proportionality in the proportions of components on the cuticle and in the PPG (y-intercept: -0.04 with 95% confidence interval of -0.30 to 0.35; slope: 1.02 with 95% confidence interval of 0.63 to 1.3).The labels of the data points correspond to the numbers in Table 1. For the regression analysis peak areas were obtained by manual integration using the GC/MS software. The peaks of some substances showed no base-line separation and were hence integrated together and treated as one peak. The total peak area of each individual extract was standardized to 100% and the relative peak areas of all peaks were calculated. Only those peaks that accounted for a mean of at least 1% of the total peak area were included in the analysis. The total peak areas of these remaining peaks were again standardized to 100%, the relative peak areas were calculated and the values normalized by log-transformation. The assumption of congruence requires direct proportionality between the samples, i.e. the slope of the regression line should not differ from 1 and the y-intercept should not differ from 0.
(TIF)

Figure S3 **Congruence between PPG/head and cuticle samples in *T. elongatus*.** A major axis regression between the relative amounts (peak area transformed; Methods analogous to the analysis of *T. boharti*) of compounds in the PPG/head and on the cuticle of *T. elongatus* females based on the eight selected peaks revealed a strong linear relationship between the relative amounts of compounds in the PPG/head samples and on the cuticle (R=0.88, P=0.004; y-intercept: 0.058 with 95% confidence interval of -0.53 to 0.93; slope: 0.97 with 95% confidence interval of -0.53 to 2.25).The labels of the data points correspond to the numbers in Table 2.
(TIF)

Figure S4 **Prey embalming by *T. elongatus* females.** Two-dimensional MDS representation of the chemical profiles of individual *T. hyalinata* control bees (squares), provisioned *T. hyalinata* bees (circles), and *T. elongatus* females (triangles) (stress value: 0.092). The ellipses depict the 95% confidence intervals. Of the five collected *T. elongatus* females we obtained chemical data of the PPG and cuticle of one individual, the head and cuticle of another individual and the cuticles of two further individuals. For a first multivariate data analysis we included all six *T. elongatus* samples into the data set. Note that the cuticle, PPG and head samples of *T. elongatus* group closely together. C, cuticle; PPG, postpharyngeal gland; H, head.
(TIF)

Table S1 **Chemical composition of the content of the postpharyngeal gland of *T. boharti* females with the additional compounds detected only in the pooled (N=10) and fractionated samples (highlighted in grey).**
(PDF)
