# Supplementary figures and images for: Morphology, Chemistry and Function of the Postpharyngeal Gland in the South American Digger Wasps Trachypus boharti and Trachypus elongatus
Source: PLoS One. 2013 Dec 6;8(12):e82780. doi: 10.1371/journal.pone.0082780 (PMC3855771; doi:10.1371/journal.pone.0082780)

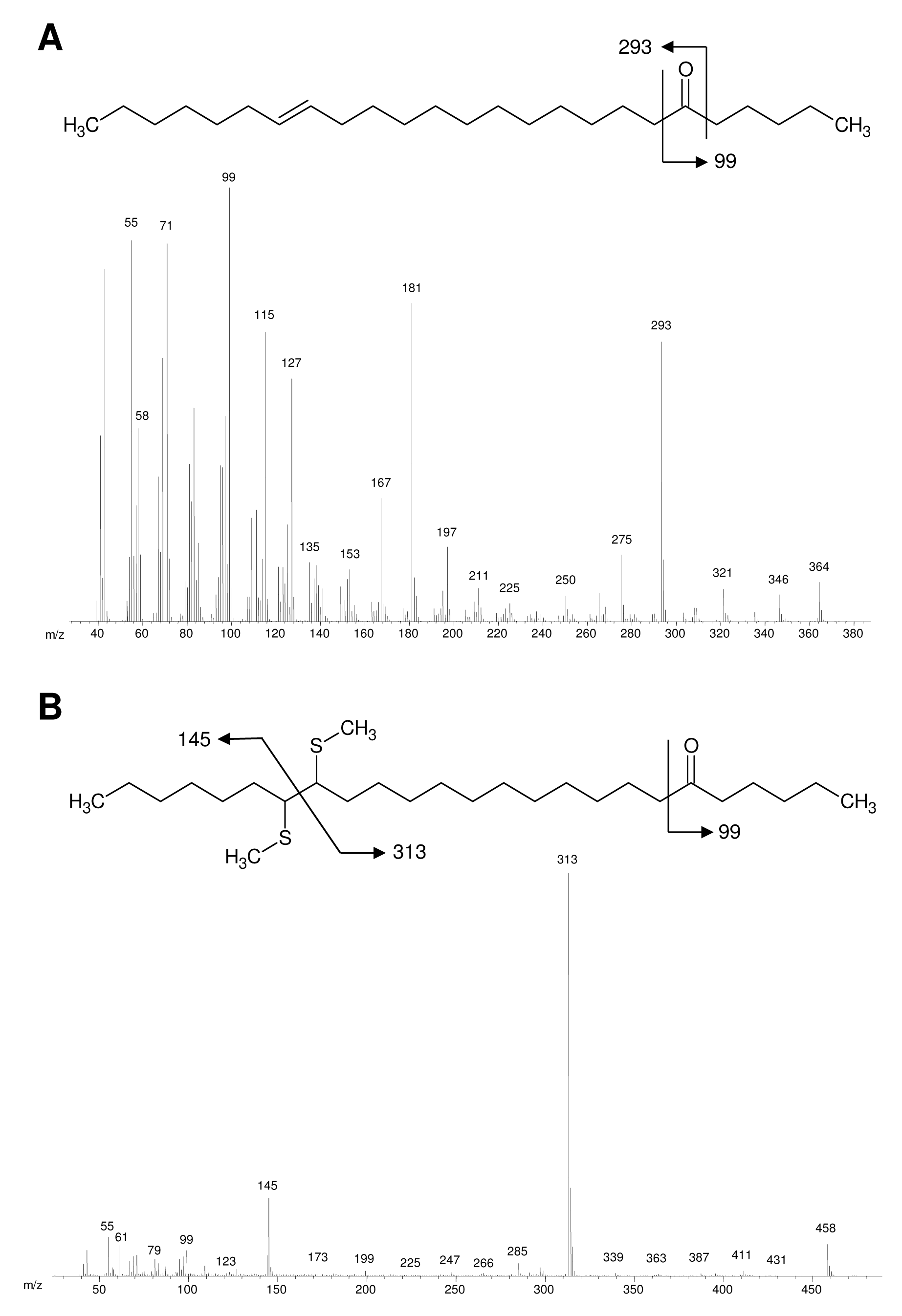

Supplement: Figure S1 — Mass spectra for (A) 18-pentacosen-6-one and (B) its DMDS adduct. (TIF) [file pone.0082780.s001.tif]

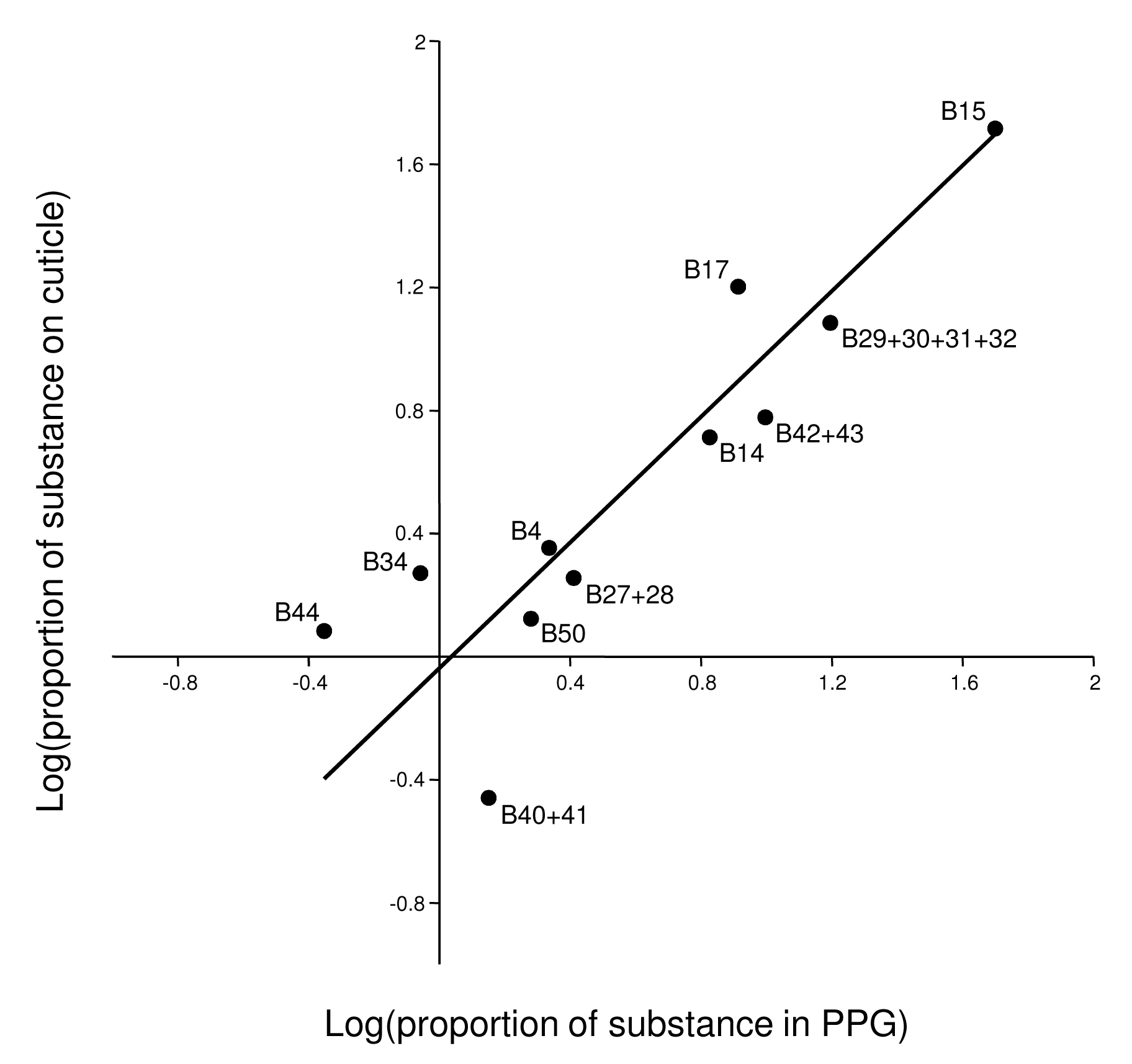

Supplement: Figure S2 — Congruence between PPG content and cuticle in T. boharti. The overall chromatographic patterns of PPG and cuticle closely resembled each other. All substances found on the cuticle were also present in the PPG. Twelve compounds were only detected in the PPG but not on the cuticle. As these substances are all minor compounds and the cuticle samples in general contained lower amounts of compounds, the twelve compounds were most probably below the detection limit in the cuticle samples. A major axis regression between the mean relative amounts of eleven selected peaks (representing 17 compounds) revealed a strong linear relationship between the compounds in the gland and the corresponding compounds on the cuticle (R=0.88, P=0.0003, N=10 individuals). Moreover, the linear regression was consistent with a direct proportionality in the proportions of components on the cuticle and in the PPG (y-intercept: -0.04 with 95% confidence interval of -0.30 to 0.35; slope: 1.02 with 95% confidence interval of 0.63 to 1.3).The labels of the data points correspond to the numbers in Table 1. For the regression analysis peak areas were obtained by manual integration using the GC/MS software. The peaks of some substances showed no base-line separation and were hence integrated together and treated as one peak. The total peak area of each individual extract was standardized to 100% and the relative peak areas of all peaks were calculated. Only those peaks that accounted for a mean of at least 1% of the total peak area were included in the analysis. The total peak areas of these remaining peaks were again standardized to 100%, the relative peak areas were calculated and the values normalized by log-transformation. The assumption of congruence requires direct proportionality between the samples, i.e. the slope of the regression line should not differ from 1 and the y-intercept should not differ from 0. (TIF) [file pone.0082780.s002.tif]

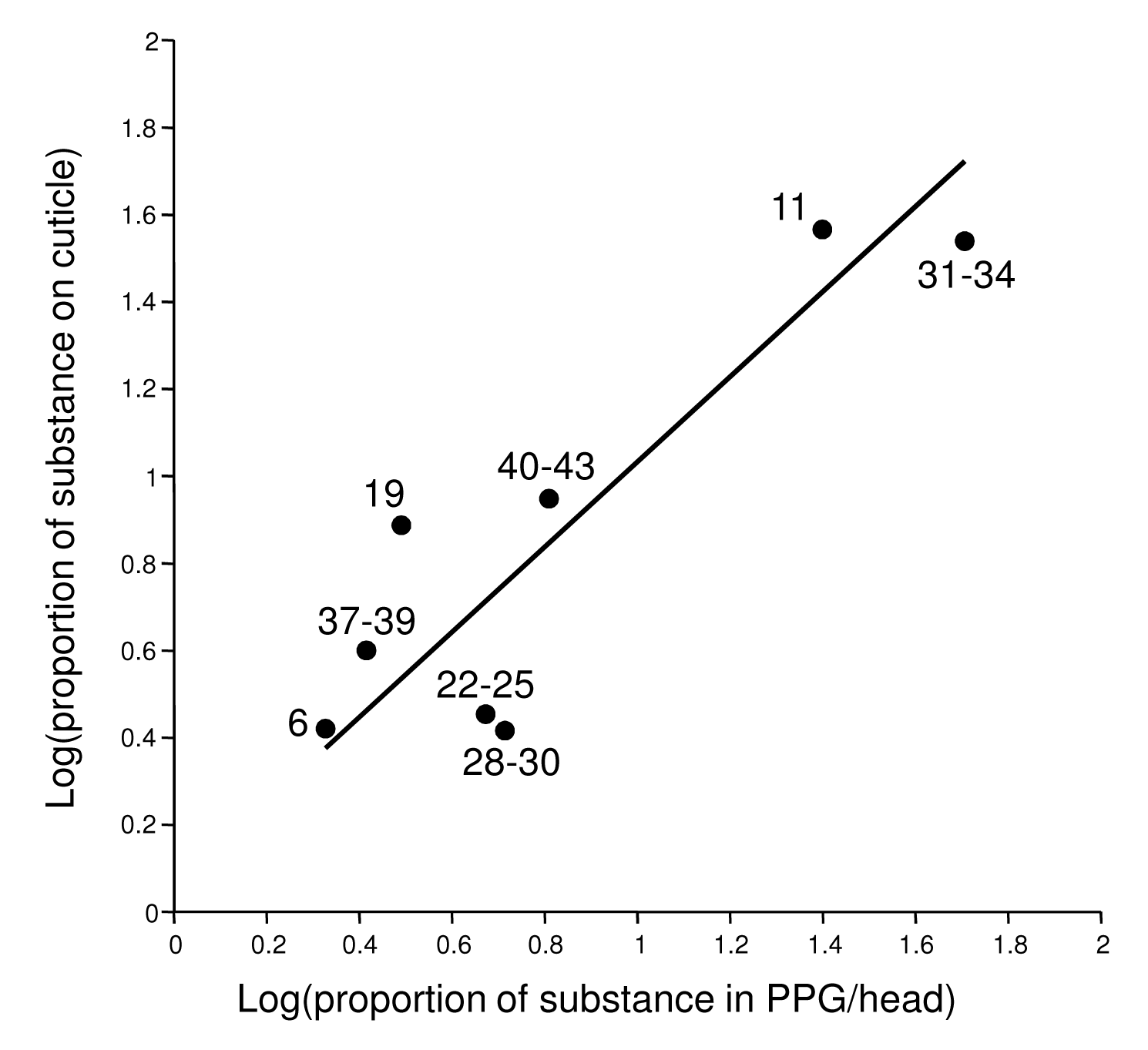

Supplement: Figure S3 — Congruence between PPG/head and cuticle samples in T. elongatus. A major axis regression between the relative amounts (peak area transformed; Methods analogous to the analysis of T. boharti) of compounds in the PPG/head and on the cuticle of T. elongatus females based on the eight selected peaks revealed a strong linear relationship between the relative amounts of compounds in the PPG/head samples and on the cuticle (R=0.88, P=0.004; y-intercept: 0.058 with 95% confidence interval of -0.53 to 0.93; slope: 0.97 with 95% confidence interval of -0.53 to 2.25).The labels of the data points correspond to the numbers in Table 2. (TIF) [file pone.0082780.s003.tif]

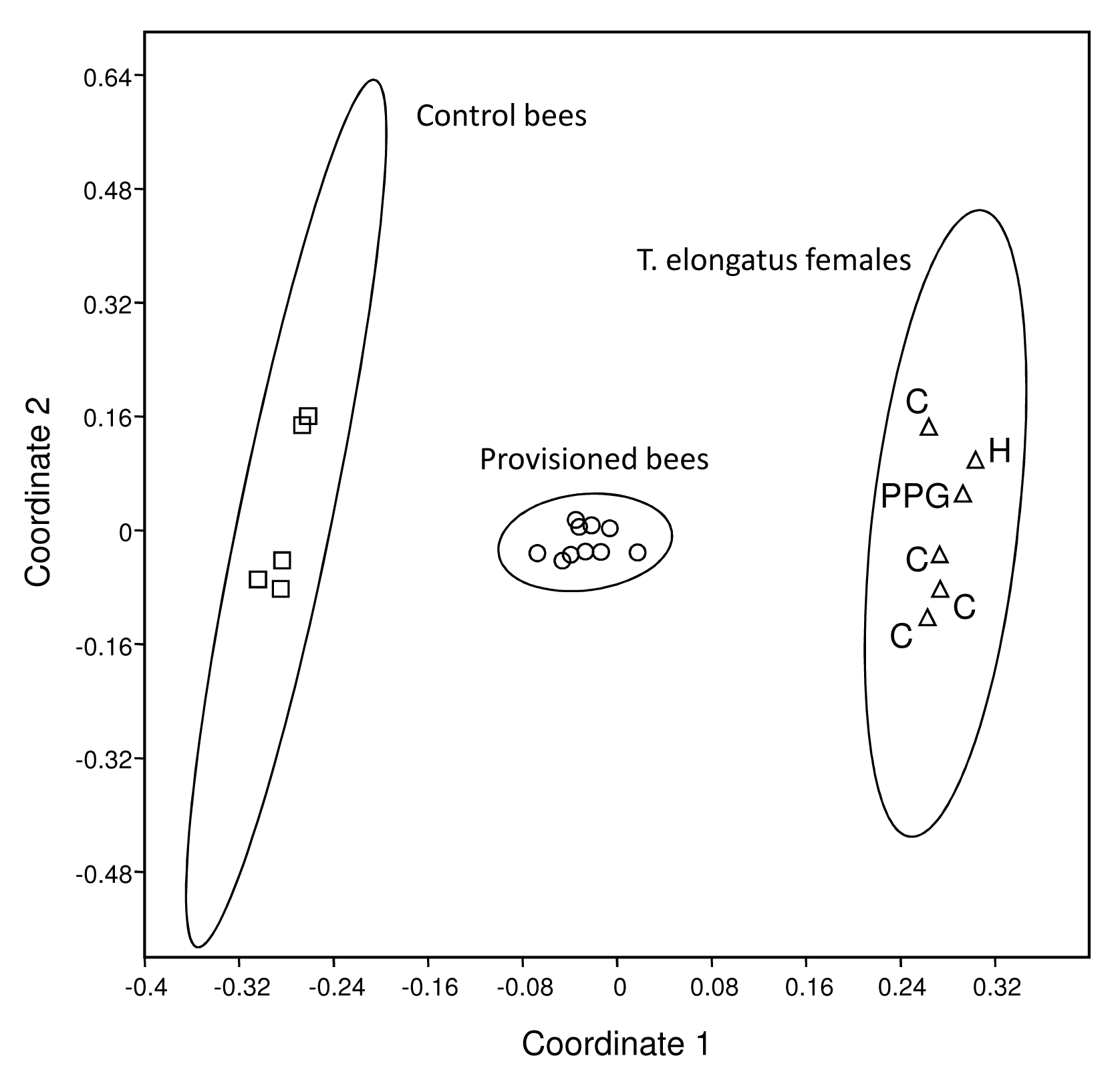

Supplement: Figure S4 — Prey embalming by T. elongatus females. Two-dimensional MDS representation of the chemical profiles of individual T. hyalinata control bees (squares), provisioned T. hyalinata bees (circles), and T. elongatus females (triangles) (stress value: 0.092). The ellipses depict the 95% confidence intervals. Of the five collected T. elongatus females we obtained chemical data of the PPG and cuticle of one individual, the head and cuticle of another individual and the cuticles of two further individuals. For a first multivariate data analysis we included all six T. elongatus samples into the data set. Note that the cuticle, PPG and head samples of T. elongatus group closely together. C, cuticle; PPG, postpharyngeal gland; H, head. (TIF) [file pone.0082780.s004.tif]
