# Supplementary material for: Morphology, Chemistry and Function of the Postpharyngeal Gland in the South American Digger Wasps Trachypus boharti and Trachypus elongatus
Source: PLoS One. 2013 Dec 6;8(12):e82780. doi: 10.1371/journal.pone.0082780 (PMC3855771; doi:10.1371/journal.pone.0082780)
Supplement: Table S1 — Chemical composition of the content of the postpharyngeal gland of T. boharti females with the additional compounds detected only in the pooled (N=10) and fractionated samples (highlighted in grey). (PDF) [file pone.0082780.s005.pdf]

Table S1: Chemical composition of the content of the postpharyngeal gland of *T. boharti* females with the additional compounds detected only in the pooled (N=10) and fractionated samples (highlighted in grey).

No.	Substance	LRI	Diagnostic ions
B1	Heneicosane	2100	296
	Docosane	2200	310
B2	9-Tricosene	2273	322; DMDS: 173, 243, 416
B3	7-Tricosene	2280	322; DMDS: 145, 271, 416
B4	Tricosane	2300	324
B5	9-Tetracosene	2273	336; DMDS: 173, 257, 430
B6	7-Tetracosene	2281	336; DMDS: 145, 285, 430
B7	Tetracosane	2400	338
B8	14-Tricosen-6-one	2454	99, 181, 265, 336; DMDS: 99, 173, 257, 430
B9	14-Tricosen-4-one	2462	71, 153, 293, 336; DMDS: 71, 173, 257, 430
B10	+ 16-Tricosen-6-one		99, 181, 265, 336; DMDS: 99, 145, 285, 430
B11	16-Tricosen-4-one	2469	71, 153, 293, 336; DMDS: 71, 145, 285, 430
B12	+ Pentacosadiene		348
B13	+ 3-Methyltetracosane		57, 323
B14	9-Pentacosene	2474	350; DMDS: 173, 271, 444
B15	7-Pentacosene	2483	350; DMDS: 145, 299, 444
	Tricosan-4-one	2488	71, 295, 338
B16	5-Pentacosene	2492	350; DMDS: 117, 327, 444
B17	Pentacosane	2500	352
B18	13-Methylpentacosane	2533	196/197 (sym.)
B19	+ 11-Methylpentacosane		168/169, 224/225
B20	5-Methylpentacosane	2550	85, 308/309
	x-Tetracosen-7-one	2563	113, 195, 265, 350; DMDS: n.d.
	17-Tetracosen-5-one	2568	85, 167, 293, 350; DMDS: 85, 145, 299, 444
B21	9-Hexacosene	2574	364; DMDS: 173, 285, 458
B22	8-Hexacosene	2577	364; DMDS: 159, 299, 458
	+ Hexacosatriene		360
B23	7-Hexacosene	2582	364; DMDS: 145, 313, 458
B24	Hexacosane	2600	366
B25	16-Pentacosen-8-one	2656	127, 209, 265, 364; DMDS: 127, 173, 285, 458
B26	16-Pentacosen-6-one	2661	99, 181, 293, 364; DMDS: 99, 173, 285, 458
B27	18-Pentacosen-8-one	2667	127, 209, 265, 364; DMDS: 127, 145, 313, 458
B29	6,9-Heptacosadiene	2671	376; DMDS: 131, 155, 203, 299, 323, 371, 407, 455, 502
B28	18-Pentacosen-6-one	2672	99, 181, 293, 364; DMDS: 99, 145, 313, 458
B30	9-Heptacosene	2675	378; DMDS: 173, 299, 472
	18-Pentacosen-4-one	2676	71, 153, 321, 364; DMDS: 71, 145, 313, 458
B31	3, 6, 9-Heptacosatriene	2680	108, 135, 318, 345, 331, 374
B32	7-Heptacosene	2683	378; DMDS: 145, 327, 472
	Pentacosan-8-one	2684	127, 267, 366
	Pentacosan-6-one	2687	99, 295, 366
	Pentacosan-4-one	2692	71, 323, 366
B33	5-Heptacosene	2694	378; DMDS: 117, 355, 472
B34	Heptacosane	2700	380
	13-Methylheptacosane	2733	196/197, 224/225, 379
	+ 11-Methylheptacosane		168/169, 252/253, 379

B35	7-Octacosene	2783	392; DMDS: 145, 341, 486
	Octacosane	2800	394
B36	18-Heptacosen-10-one	2861	155, 237, 265, 392; DMDS: 155, 173, 313, 486
B37	+ 18-Heptacosen-8-one		127, 209, 293, 392; DMDS: 127, 173, 313, 486
B38	20-Heptacosen-10-one	2869	155, 237, 265, 392; DMDS: 155, 145, 341, 486
B39	+ 20-Heptacosen-8-one		127, 209, 293, 392; DMDS: 127, 145, 341, 486
B40	6,9-Nonacosadiene	2874	404; DMDS: 131, 155, 203, 327, 351, 399, 435, 483, 530
B41	9-Nonacosene	2876	406; DMDS: 173, 327, 500
	20-Heptacosen-4-one	2878	71, 349, 392; DMDS: 71, 145, 341, 486
B42	3, 6, 9-Nonacosatriene	2882	108, 135, 346, 359, 373, 402
	Heptacosan-10-one	2886	155, 267, 394
	+ Heptacosan-8-one		127, 295, 394
B43	7-Nonacosene	2888	406; DMDS: 145, 355, 500
	Heptacosan-4-one	2894	71, 351, 394
	5-Nonacosene	2895	406; DMDS: 117, 383, 500
B44	Nonacosane	2900	408
B45	7-Triacontene	2985	420; DMDS: 145, 369, 514
	Triacontane	3000	422
B46	22-Nonacosen-12-one	3071	183, 265, 420; DMDS: 171, 145, 369, 514
B47	+ 22-Nonacosen-10-one		155, 237, 293, 420; DMDS: 155, 145, 369, 514
	x-Nonacosen-6-one	3078	99, 349, 420
B48	Hentriacontadiene	3078	432; DMDS: n.d.
B49	+ 9-Hentriacontene		434; DMDS: 173, 355, 528
	Nonacosan-12-one	3087	171, 267, 422
	+ Nonacosan-10-one		155, 295, 422
B50	7-Hentriacontene	3087	434; DMDS: 145, 383, 528
B51	Hentriacontane	3100	436
	Tritriacontene	3272	462
	Tritriacontene	3279	462

LRI = linear retention index (calculated in relation to *n*-alkanes in an alkane standard) on the RH-5ms+ column

sym = symmetric molecule, reduced number of diagnostic ions;

DMDS = diagnostic ions of the respective DMDS adducts;

n.d. = not detected.
